# PERSPECTIVES TOWARD ADVANCE CARE PLANNING IN FAMILY CAREGIVERS OF PERSONS WITH DEMENTIA: Q METHODOLOGY STUDY

**DOI:** 10.1093/geroni/igad104.2976

**Published:** 2023-12-21

**Authors:** KyungAh Cho, Myoung Hwan Shin, Jung-Ah Lee, Seongkum Heo, Sang Yi Baek, Jisun Yang, SeongHu Park, JinShil Kim

**Affiliations:** Gachon university, Incheon, Republic of Korea; Sookmyung Women’s University, Seoul, Republic of Korea; University of California-Irvine, Irvine, California, United States; Mercer University, Georgia Baptist College of Nursing, Atlanta, Georgia, United States; Gachon university, Incheon, Republic of Korea; Gachon university, Incheon, Republic of Korea; Sungshin Women’s University, Seoul, Republic of Korea; Gachon university, Incheon, Republic of Korea

## Abstract

**Background:**

Family caregivers’ perspectives toward advance care planning (ACP) is critically important to reflect end-of-life wishes of people with dementia (PWD). Purpose: To explore the ACP perspectives among family caregivers of PWD using a Q methodology. Methodology: Q sample with statements regarding the characteristics of caregivers’ perspectives toward ACP was constructed. P sample of 25 caregivers completed each grid with a statement for Q sorting table. Data analysis was done using the PQ Method program (Ver. 2.35).

**Results:**

Three factors emerged through a Varimax rotation and explained 53% of the total variance. Factor 1 (n = 12) (General cases: Planned preparation of pre-care planning (PPP) and understanding and thoughtfulness of the behaviors of PWD) consisted of caregivers who knew the importance of PPP and willingly tried to understand and manage dementia. Factor 2 (n = 7) (Severe cases: Unplanned preparation of PPP and feeling of responsibility and burden for management of PWD) consisted of caregivers who did not have PPP and had concerns and anxiety about management of PWD. Factor 3 (n = 5) (Mild cases: Optimistic preparation of PPP and proudness for caring PWD) consisted of caregivers who were optimistic about dementia care, were proud of their duty of caring PWD, and thought meeting patients’ needs and caring them as valuable.

**Conclusions:**

Caregivers of PWD had different ACP perspectives based on the severity of cognitive impairment in patients. Clinicians should consider the levels of patients’ cognitive impairment to provide effective ACP education to caregivers of PWD.

